# Level of screening for and vaccination against hepatitis B among healthcare workers in the Eastern Democratic Republic of the Congo: a public health concern

**DOI:** 10.1016/j.infpip.2022.100226

**Published:** 2022-06-24

**Authors:** F.K. Sikakulya, D.K. Munyambalu, S.B. Mambo, A.K. Mutsunga, S.F. Djuma, P.A. Djuna, E. Ndiwelubula, W.A. Ngavo, S.M. Sahika, Patrick Kumbowi Kumbakulu, Kalima Nzanzu Adelard, T.A. Shindano

**Affiliations:** a Department of General Surgery, Kampala International University, Western Campus, Bushenyi, Uganda; b Faculty of Medicine, Université Catholique du Graben, Butembo, Democratic Republic of the Congo; c Department of Internal Medicine, Kampala International University, Western Campus, Bushenyi, Uganda; d Youth Alliance for Reproductive Health, Goma, Democratic Republic of the Congo; e Department of Public Health, School of Allied Health Sciences, Kampala International University, Western Campus, Bushenyi, Uganda; f Department of Internal Medicine, Hôpital Provincial Général de Référence de Bukavu (HPGRB), Bukavu, Democratic Republic of the Congo; g Faculty of Medicine, Université Catholique de Bukavu (UCB), Bukavu, Democratic Republic of the Congo; h University of Kindu, Kindu, Maniema, Democratic Republic of the Congo; i Department of Paediatrics and Child Health, Kampala International University, Western Campus, Bushenyi, Uganda

**Keywords:** Hepatitis B, Healthcare workers, Screening and vaccination, Eastern DR Congo

## Abstract

**Background:**

In low resource settings in sub-Saharan Africa healthcare workers (HCW) have a high risk of contracting hepatitis B infection. Vaccination of HCWs is to protect them from acquisition of hepatitis B from patients.

**Objective:**

To evaluate the hepatitis B virus (HBV) serological and vaccination status of HCWs in the Butembo Antenna in the Eastern Democratic Republic of Congo (DR Congo) and to investigate the factors influencing hepatitis screening and vaccination.

**Methods:**

A cross-sectional study using a structured questionnaire was carried out from 1^st^ to 30^th^ April 2021 among consenting HCWs in Butembo (≥18 years of age). Data was analysed using SPSS version 23.

**Results:**

Of 373 participants, 178 (47.7%) had already been screened for HBV. Screening was more likely for HCWs in a rural or publicly owned facility (*P*<0.05). A total of 25 (6.7%) HCWs were fully vaccinated against HBV; the factors associated with full vaccination were: prior screening for HBV (odds ratio: 9.03 (2.51–38.61), *P*<0.0001), prior knowledge of the value of post-exposure prophylaxis (odds ratio 12.9 (2.89–80.44), *P*=0.0004), prior knowledge of hepatitis B vaccine benefits (adjusted odds ratio: 4.54 (1.66–13.05), *P*=0.002) and prior exposure to hepatitis B infection (adjusted odds ratio: 2.61 (1.08–6.39), *P*=0.039).

**Conclusion:**

Screening and vaccination rates of HCWs for HBV are extremely low, and not high enough to prevent the spread of this serious illness and its complications. There is a dire need to increase vaccination rates among HCWs in Eastern DR Congo. The DRC government should issue vaccination against HBV as a recommendation at the national level.

## Introduction

Low- and middle-income countries account for 96% of the persons globally living with hepatitis B, yet access to testing and treatment for the condition is limited in these countries [1]. The global burden of hepatitis B virus (HBV) is enormous, with 257 million persons chronically infected, resulting in more than 880,000 deaths per year worldwide [1,2].

Higher prevalence is encountered in low-income countries, including those of sub-Saharan Africa where HBV infection is hyperendemic [3]. In 2015, global coverage with a third dose of hepatitis B vaccine reached 84%, but there were lower vaccination rates in European, Eastern Mediterranean and African regions [1,4]. Healthcare workers (HCWs) are considered a high-risk group for HBV infection due to occupational exposure to blood-borne pathogens. Worldwide, approximately 2 million HCWs are infected with HBV through needle stick and other sharps injuries [4]. Previous studies in Africa found high HBV infection and exposure rates (roughly 10%) in HCWs in South Africa and Nigeria [5]. HBV infections in healthcare settings usually result from both needle stick injuries and failure to adhere to universal infection prevention precautions. The evidence available suggests that almost half of HCWs in Africa are occupationally exposed to body fluids annually [6].

The low uptake of HBV vaccination services among HCWs in sub-Saharan Africa has been attributed to the cost of the vaccine and lack of awareness of its availability [7]. This is coupled with limited evidence of screening and uptake of hepatitis B vaccination among HCWs, and a knowledge gap on the factors that may influence HBV screening and completion of the hepatitis B vaccination schedule [4]. A study carried out in Uganda found that a quarter of HCWs had never been screened for HBV and less than four in 10 were not fully vaccinated, thus not meeting the Uganda Ministry of Health recommendation that all HCWs should be screened [7]. However, in Tanzania it was observed that only one in three HCWs at the national referral hospital had undergone full HBV vaccination, despite high levels of awareness and the near complete acceptance of the vaccine and its importance by HCWs [8].

In the Democratic Republic of the Congo (DRC), health and education systems have been disrupted by multiple socio-political instabilities. Although its prevalence of HBV infection is among the highest in the world [9], the overall risk of these infections to HCWs is largely unknown [10]. A survey conducted in Bukavu, a town in South Kivu DRC, revealed that HCWs had a low level of knowledge about HBV and only 3.8% of vaccinated HCWs had received the complete vaccination schedule of three vaccine doses [10].

The Centers for Disease Control and Prevention (CDC) recommends that high-risk groups such as HCWs should be screened for and vaccinated with three doses of HBV vaccine [7,11]. HBV screening and diagnosis are vital for access to appropriate prevention, care and treatment services [7]. The DRC had endorsed ‘the call of RABAT’ in 2009 to implement concrete strategies for the reduction of HBV prevalence [10]. These strategies include the promotion of preventive actions, such as reduction in mother-to-child HBV transmission and mandatory vaccination of all HCWs (i.e., medical doctors, students, nurses, laboratory students and laboratory technicians) [11,12].

Notwithstanding the several recent epidemics of Ebola, COVID-19 and cholera, there are no published data on the serological and vaccination status of HBV infection among HCWs in the Eastern region of the DRC. Therefore, the aim of this study was to determine the serological and vaccination status of HCWs in the Butembo Antenna in Eastern DRC against HBV, and the factors that might influence their screening and immunization.

## Methods

### Study design and setting

This was a cross-sectional study conducted in the main referral hospitals of the Butembo Health Antenna, a network of hospitals that collaborate and support each other to provide an integrated healthcare service in the northern part of the North Kivu province of the Eastern DRC. The Antenna has 17 health zones served by referral hospitals, which are localized in Beni and Butembo towns and throughout the Beni and Lubero territories. In addition, there are two teaching hospitals: Cliniques Universitaires du Graben of the Universite Catholique du Graben and Matanda Hospital, both localated in Butembo Town. These hospitals were selected because they are the key points of treatment in the Antenna and receive referrals from all the health centres within the Butembo Antenna in Eastern DRC. Because these well-equipped hospitals care for many severely ill patients, many of their HCWs are at risk of exposure to HBV and other infectious diseases.

### Source and study participants

Data were collected from randomly selected HCWs who consented to participate to the study and worked in one of 18 pre-selected hospitals in the Butembo Antenna in Eastern DRC. The number of participants selected from each hospital was according to the number of HCWs they employed, and staff availability during the data-collection period. The sample size was determined using the Kish Leslie formula. Considering the completion of HBV immunization status of 57.8% from a study carried out in Uganda [8] at 95% level of confidence and a margin of error of 0.05, a minimum sample size of 375 HCWs was obtained. This was increased to 413, based on the assumption that a questionnaire non-completion rate could be as high as 10%.

### Data collection and instrument

Data were collected for a period of one month from 1^st^ April to 30^th^ April 2021. Written informed consent was obtained from each participant before completing the structured questionnaire. The study tool was designed as a multiple-choice questionnaire with ‘Yes’ and ‘No’ answers. The questionnaire was composed of 20 items based on a published tool validated by Ssekamatte *et al.* on Ugandan HCWs [7], which focused on several key constructs: eight questions related to socio-demographic characteristics (location of health facility, sex, age in years, marital status, HCW cadre, hospital category, years of experience as HCW, ownership of facility); seven questions related to knowledge about HBV infection and serological status of the participants; two questions related to attitude of HCWs regarding HBV infection and three questions of practices of HCWs regarding measures to prevent HBV transmission (including vaccination).

### Statistical methods and data analysis

Data were refined and entered into Microsoft Excel and exported into SPSS version 23 for statistical analysis. Descriptive analyses such as frequencies, proportions, and means (where appropriate) were performed for HCW socio-demographic characteristics, and their knowledge, attitudes and practice regarding HBV. Numeric continuous variables were compared using Student's *t*-test and the unadjusted odds ratios of categorical variables were compared using Chi-squared analysis with Yates' continuity correction. The *P*-value for statistical significance was 0.05.

### Ethical considerations

This study was given ethical approval from the ‘Comité Ethique du Nord-Kivu’ in DRC (001/TEN/CENK/2021). Permission to access health facilities was obtained from the management of different health facilities and relevant local health authorities and consent was obtained from HCWs before participation in the study.

## Results

### Socio-demographic characteristics of the participants

The final study population was the 373 (90.3%) of the 413 HCW who were asked to complete the questionnaire. Their mean age was 36.8 (standard deviation 10.7) years and 200 (53.6%) were female; 178 (47.7%) had been screened for HBV and 25 (6.7%) were fully vaccinated. Most participants were nurses, married and worked in an urban publicly owned health facility (Table I).

**Table I:** Socio-demographic characteristics of the study participants

	Total		Screened		Unscreened		Vaccinated		Unvaccinated	
*N*	373		178 (47.7%)		195		25 (6.7%)		349	
Age (years)	36.8 (SD 10.7)		38.4 (SD 11.7)		36.5 (SD 10.5)		35.8 (SD 9.7)		36.9 (SD 10.8)	
Experience (years)	10.4 (SD 9.5)		10.2 (SD 8.9)		10.5 (SD 10.1)		8.8 (SD 6.9)		10.5 (SD 9.17)	
Female sex	200	(53.6%)	98	(49.0%)	102	(51.0%)	17	(8.5%)	183	(91.5)
Male sex	173	(46.4%)	80	(44.9%)	93	(47.7%)	8	(32.0%)	166	(47.6%)
Married	238	(63.8%)	128	(71.9%)	110	(56.4%)	12	(48.0%)	227	(65.0%)
Unmarried	135	(36.2%)	50	37.0%	85	(63.0%)	13	(9.6%)	122	(90.4)
Public facility	230	(61.7%)	128	(71.9%)	102	(52.3%)	21	(84.0%)	210	(60.2%)
Private facility	143	(38.3%)	50	(35.0%)	93	(65.0%)	4	(2.8%)	139	(97.2%)
Urban facility	224	(60.1%)	91	(40.6%)	133	(59.4%)	9	(4.0%)	215	(96%)
Rural facility	149	(39.9%)	87	(48.9%)	62	(31.8%)	16	(64.0%)	133	(38.1%)
Nurses	216	(57.9%)	99	(55.6%)	117	(60.0%)	15	(60.0%)	201	(57.6%)
Doctors	66	(17.7%)	40	(22.5%)	26	(13.3%)	5	(20.0%)	61	(17.5%)
Non-clinical HCWs	53	(14.2%)	14	(7.9%)	39	(20.0%)	3	(12.0%)	50	(14.3%)
Lab technicians	28	(7.5%)	25	(14.0%)	13	(6.7%)	2	(8.0%)	36	(10.3%)

HCW, healthcare worker; SD, standard deviation.

### Screening and vaccination according to HCWs' socio-demographic characteristics

Participants who worked in a rural or public facility or were laboratory technicians were more likely to be screened for HBV. HCWs who had been screened were nine times more likely to be vaccinated than those who had not been screened. The only other factors identified that significantly increased the chance of vaccination were working in a rural or public facility (Table II).

**Table II:** Association between screening and vaccination with healthcare workers' socio-demographic characteristics

Variable	Odds ratio (95% CI)		Chi-squared	*P*
Being screened				
Public vs private	2.33	(1.48–3.68)	14.31	<0.0001
Laboratory technician vs non-clinical HCW	5.36	(1.98–14.80)	12.45	<0.0001
Clinical vs non-clinical HCW	2.93	(1.47–5.91)	10.27	<0.0001
Rural vs urban facility	2.05	(1.32–3.20)	10.62	<0.0001
Married vs unmarried HCW	1.98	(1.26–3.12)	9.02	<0.0001
Male vs female HCW	0.90	(0.58–1.37)	0.18	0.67
Being vaccinated				
Screened vs not screened	9.03	(2.51–38.61)	15.74	<0.0001
Rural vs urban	2.87	(1.16–7.27	5.43	0.02
Public vs private facility	3.49	(1.10–12.29	4.69	0.03
Married vs unmarried HCW	0.50	(0.21–1.21	2.21	0.14
Male vs female HCW	0.52	(0.20–1.32	1.65	0.20
Lab technician vs non-clinical HCW	0.93	(0.10–7.32)	0.15	0.70
Clinical vs non-clinical HCW	1.23	(0.33–5.37)	0.00	0.98

CI, confidence interval; HCW, healthcare worker.

### HCW's knowledge, attitudes and practice

Although most HCWs (94.1%) believed HBV infection could be controlled in their work environment, HBV screening in their hospital was only available to 25.7% of them and fewer than half (43.4%) knew about post-exposure prophylaxis (Table III).

**Table III:** Distribution of healthcare workers' knowledge, attitudes, and practice

HCW knowledge, attitudes and practice	Total (*N* = 373)		Screened		Vaccinated	
Knowledge						
HCW knows HBV infection can be transmitted by carriers	296	(79.4%)	162	(54.7%)	24	(8.1%)
HCW knows HBV carries a high risk of morbidity	231	(61.9%)	140	(60.6%)	19	(8.2%)
HCW knows they are at high risk of HBV infection	310	(83.1%)	171	(55.2%)	24	(7.7%)
HCW knows the modes of HBV transmission	302	(81.0%)	169	(56.0%)	24	(7.9%)
HCW knows how to prevent HBV infection	298	(79.9%)	158	(53.0%)	22	(7.4%)
HCW knows about post-exposure prophylaxis	162	(43.4%)	97	(59.9%)	19	(11.7%)
HCW knows the benefits of the hepatitis B vaccine	187	(50.1%)	108	(57.8%)	23	(12.3%)
Attitudes						
HCW believes HBV infection can be controlled in their environment	351	(94.1%)	171	(48.7%)	24	(6.8%)
HCW thinks they should be vaccinated	178	(47.7%)	78	(43.8%)	17	(9.6%)
Practice						
HCW knows their department receive patients with confirmed hepatitis B	289	(77.5%)	147	(50.9%)	20	(6.9%)
HCW knows he/she has been exposed to hepatitis B infection	128	(34.3%)	85	(66.4%)	14	(10.9%)
HBV screening available in their hospital	96	(25.7%)	52	(54.2%)	8	(8.3%)

HBV, hepatitis B virus; HCW, healthcare worker.

### Screening and vaccination status with knowledge, attitudes, and practice

A positive response to the questions about HBV knowledge were significantly associated with the likelihood of a HCW being screened; the strongest association was knowing how hepatitis B is transmitted and the risk of infection (χ^2^-values >41). The only attitudes and practices associated with screening were exposure to HBV at work, and the belief that vaccination for HCWs should be optional (Table IV). Only knowing about post-exposure prophylaxis (χ^2^ 10.19), the benefits of vaccination (χ^2^ 17.03), and exposure to hepatitis at work (χ^2^ 4.61) were significantly associated with vaccination status (Table IV).

**Table IV:** Association between screening and vaccination status with knowledge, attitudes and practice of participants

Knowledge, attitudes and practice	Odds ratio (95% CI)		Chi-squared	P
Participants who had been screened				
Knowledge				
HBV infection can be transmitted by carriers	4.61	(2.45–8.76)	26.89	<0.0001
HBV carries a high risk of morbidity	4.21	(2.61–6.82)	39.03	<0.0001
Know they are at high risk of HBV infection	11.48	(4.58–30.55)	41.05	<0.0001
Know the mode of HBV transmission	8.75	(4.02–19.64)	41.45	<0.0001
Know how to prevent HBV infection	3.10	(1.72–5.65)	15.64	0.0001
Knows about post-exposure prophylaxis	2.40	(1.54–3.73)	16.11	0.0001
Know the benefits of the hepatitis B vaccine	2.27	(1.46–3.51)	14.33	0.0002
Attitudes				
Believes hepatitis infection can be controlled in their environment	2.04	(0.76–5.66)	1.71	0.19
Vaccination among HCWs should be mandatory	0.74	(0.48–1.14)	1.79	0.18
Vaccination among HCW should be optional	2.05	(1.28–3.29)	9.35	0.002
Practice				
Their department receives patients with confirmed hepatitis B	1.77	(1.04–3.01)	4.54	0.03
Have been exposed to hepatitis B infection	3.23	(2.01–5.19)	26.14	<0.0001
Hepatitis B screening available in their hospital	1.42	(0.87–2.32)	1.82	0.18
Participants who had been vaccinated				
Knowledge				
HBV infection can be transmitted by carriers	6.71	(0.94–135.31)	3.51	0.06
HBV carries a high risk of morbidity	2.03	(0.74–5.84)	1.66	0.2
Know they are at high risk of HBV infection	5.10	(0.71–103.21)	2.18	0.14
Know the mode of HBV transmission	6.04	(0.85–122.05)	2.95	0.09
Know how to prevent HBV infection	1.91	(0.52–8.27)	0.62	0.43
Know about post-exposure prophylaxis	4.54	(1.66–13.05)	10.19	0.001
Know the benefits of the hepatitis B vaccine	12.90	(2.89–80.44)	17.03	0.0004
Attitudes				
Believe hepatitis infection can be controlled in their environment	1.54	(0.20–32.04)	0.00	0.98
Vaccination among HCWs should be mandatory	2.47	(0.97–6.42)	3.59	0.06
Vaccination among HCWs should be optional	0.54	(0.17–1.58)	0.98	0.32
Practice				
Their department receives patients with confirmed hepatitis B	1.17	(0.40–3.70)	0.00	0.95
Have been exposed to hepatitis B infection	2.61	(1.08–6.39)	4.61	0.03
Hepatitis B screening available in their hospital	1.39	(0.53–3.56)	0.25	0.61

CI, confidence interval; HBV, hepatitis B virus; HCW, healthcare worker.

## Discussion

In this sample of HCWs working in a region of the DRC with a high prevalence of HBV infection, screening and vaccination rates are low. Married HCWs working in a rural or publicly owned facility were more likely to be both screened and vaccinated, and HCWs who had been screened were nine-times more likely to be vaccinated. HCWs who had been screened were more knowledgeable about hepatitis than those who were not screened, and vaccination was significantly more likely if HCWs were exposed to hepatitis at work, knew about post-exposure prophylaxis and the benefits of vaccination.

We found that 47.7% of HCWs had been screened for HBV, a lower screening rate than in Uganda (75.2%) [7] but higher than in Tanzania, where only 9% of HCWs were aware of their HBV status [13]. Although the 6.7% HCW vaccination rate we observed is higher than the 3.8% in HCW in Bukavu in South Kivu DRC [10], it is extremely low than in many other countries in Africa. Out of a sample of 348 HCWs in Tanzania, 198 (56.9%) were fully vaccinated [14], of 286 HCWs in Ethiopia 58.6% were fully vaccinated [15]. HCWs in Ghana, a provincial hospital in Kenya and a University Hospital in Ethiopia reported lower coverages of 53%, 42% and 28%, respectively [[16], [17], [18]].

Screening for HBV infection is the gateway to access for both prevention and treatment services and is a crucial component of an effective response to the hepatitis B epidemic [19]. Surprisingly, we found HCWs in rural settings were more likely to be screened and vaccinated. In Uganda and China, urban HCWs were more likely to be screened [7,20] possibly because of the greater availability of screening kits. The low screening and vaccination rates we observed could be due to the regular disruption of the health system in the eastern DRC due to socio-political instabilities (including war) [9]. Despite these issues, we found that HCWs who knew they were at high risk of HBV infection, understood its mode of transmission, and the benefit of vaccination were those most likely to have been screened for HBV. However, it is unclear whether more knowledgeable HCWs sought out screening, or whether the screening process made them more knowledgeable. Nevertheless, as other studies of African HCWs have shown [14,15,21], vaccination against HBV was more likely in HCWs who were more knowledgeable about the virus and its dangers. A study carried out in a Nigerian teaching hospital has shown that the provision of free vaccine increases its uptake by HCWs to over 90% [22]. This strategy should be encouraged and promoted by Public Health programmes in lower-resourced and conflict areas such as the Eastern DRC.

This is the only study of HBV screening and vaccination rates of HCW in the Eastern DRC since it became a conflict zone 30 years ago. Therefore, it cannot be assumed that our findings are applicable to all HCWs in the DRC. It is a small study and our findings may suffer from social desirability bias as HCWs may have been motivated to report favourable health access and utilization. This cross-sectional study only provides associations with HBV screening and vaccination, and the causality of the factors should not be inferred. In addition to that, knowledge of HCWs was assessed using self-reported questions.

In conclusion, HBV screening level is low and vaccination level of HCW in the Eastern part of the DR Congo is extremely low and not high enough to prevent the spread of this serious illness and its complications. Our data suggest that knowledge improves HBV screening, which enhances the likelihood of vaccine uptake. This may be an important factor, in addition to vaccination, for the prevention of transmission of the disease to HCWs and their patients. Vaccination against hepatitis B should be recommended for HCWs in Eastern DRC. The DRC government should issue vaccination against HBV as a recommendation at the national level.

## References

[1] WHO (2017). https://apps.who.int/iris/bitstream/handle/10665/255016/9789241565455-eng.pdf.

[2] Revill P.A., Tu T., Netter H.J., Yuen L.K.W., Locarnini S.A., Littlejohn M. (2020 Oct). The evolution and clinical impact of hepatitis B virus genome diversity. Nat Rev Gastroenterol Hepatol.

[3] Kengibe P.Y., Makulo J.R., Nlandu Y.M., Lepira F.B., Sumaili E.K., Bukabau J.B. (2019). Response to single dose hepatitis B vaccine in Congolese non-HIV hemodialysis patients: a prospective observational study. Pan Afr Med J.

[4] Qin Y., Li B., Zhou Y., Zhang X., Li L., Song B. (2018). Prevalence and associated knowledge of hepatitis B infection among healthcare workers in Freetown, Sierra Leone. BMC Infect Dis.

[5] Lungosi M.B., Muzembo B.A., Mbendi N.C., Nkodila N.A., Ngatu N.R., Suzuki T. (2019). Assessing the prevalence of hepatitis B virus infection among health care workers in a referral hospital in Kisantu, Congo DR: a pilot study. Ind Health.

[6] Auta A., Adewuyi E.O., Tor-anyiin A., Aziz D., Ogbole E., Ogbonna O. (2017). Health-care workers ’ occupational exposures to body fluids in 21 countries in Africa: systematic review and meta-analysis. Bull World Health Organ.

[7] Ssekamatte T., Mukama T., Kibira S.P.S., Ndejjo R., Bukenya J.N., Kimoga Z.P.A. (2020 Jul 9). Hepatitis B screening and vaccination status of healthcare providers in Wakiso district, Uganda. PLoS One.

[8] Aaron D., Nagu T.J., Rwegasha J., Komba E. (2017 Dec 20). Hepatitis B vaccination coverage among healthcare workers at national hospital in Tanzania: how much, who and why?. BMC Infect Dis.

[9] Ilumbulumbu M., Ketha J., Tshimanga V., Bunduki G., Valimungighe M., Kitamwivirirwa T. (2018). High Prevalence of Transfusion-Transmissible Infections among Volunteer Blood Donors in Rural Area of Eastern Democratic Republic of the Congo (D.R.C). Arch Curr Res Int.

[10] Amrani N. (2017). The Declaration of Rabat: An update. Arab J Gastroenterol.

[11] Centers for Disease Control and Prevention (CDC) (2012). Updated CDC recommendations for the management of hepatitis B virus-infected health-care providers and students. MMWR Recomm Rep (Morb Mortal Wkly Rep).

[12] Shindano T.A., Kabinda J.M., Mitashi P., Horsmans Y. (2018). Hepatitis B virus infection in the Democratic Republic of Congo: a systematic review of prevalence studies (2000–2016). J Public Health.

[13] Debes J.D., Kayandabila J., Pogemiller H. (2016). Knowledge of hepatitis B transmission risks among health workers in Tanzania. Am Soc Trop Med Hyg.

[14] Aaron D., Nagu T.J., Rwegasha J., Komba E. (2017). Hepatitis B vaccination coverage among healthcare workers at national hospital in Tanzania: how much, who and why?. BMC Infect Dis.

[15] Ayalew M.D., Horsa B.A. (2017). Hepatitis B vaccination status among health care workers in a tertiary hospital in Ethiopia. Hepat Res Treat.

[16] Ansa G.A., Ofori K.N.A., Houphouet E.E., Amoabeng A.A., Sifa J.S., Amenuveve C.K. (2019 Jun 10). Hepatitis B vaccine uptake among healthcare workers in a referral hospital. Accra. Pan Afr Med J..

[17] Mbaisi E.M., Ng'ang'a Z., Wanzala P., Omolo J. (2013). Prevalence and factors associated with percutaneous injuries and splash exposures among health-care workers in a provincial hospital, Kenya, 2010. Pan Afr Med J.

[18] Biset A.M., Adugna H.B. (2017). Hepatitis B vaccination status among health care workers in a tertiary hospital in Ethiopia. Hepat Res Treat.

[19] WHO (2017). https://www.who.int/hepatitis/publications/HEP17001_WEB11.pdf?ua=1.

[20] Yuan Q., Wang F., Zheng H., Zhang G., Miao N., Sun X. (2019). Hepatitis B vaccination coverage among health care workers in China. PLoS One.

[21] Alege J.B., Gulom G., Ochom A., Kaku V.E. (2020). Assessing level of knowledge and uptake of hepatitis B vaccination among health care workers at Juba Teaching Hospital, Juba City, South Sudan. Adv Prev Med.

[22] Fatusi A.O., Fatusi O.A., Esimai A.O., Onayade A.A., Ojo O.S. (2000). Acceptance of hepatitis B vaccine by workers in a Nigerian teaching hospital. East Afr Med J.

